# Social Media and E-Cigarette Use Among Adolescents and Young Adults: A Systematic Review of the Role of Influencers and Platforms in Promoting Vaping Culture

**DOI:** 10.1192/j.eurpsy.2025.782

**Published:** 2025-08-26

**Authors:** K. Jafari, N. Joshaghani, A. Wascher, S. Gunturu

**Affiliations:** 1 Psychiatry, BronxCare, New York; 2 American University of the Caribbean, Miramar, United States

## Abstract

**Introduction:**

The widespread use of electronic cigarettes (e-cigarettes) among adolescents and young adults has become a significant public health concern. With the rise of social media, exposure to content promoting e-cigarette use, including endorsements by influencers, has become increasingly pervasive. This overlap between social media and vaping culture may contribute to the normalization and growing use of e-cigarettes among this vulnerable population.

**Objectives:**

This article aims to systematically examine the influence of social media use and endorsements by influencers on e-cigarette use among adolescents and young adults, identifying patterns of exposure and consumption across various platforms and providing insights for future prevention and intervention strategies.

**Methods:**

This systematic review aims to explore the impact of social media use and influencers on e-cigarette use among adolescents and young adults. A PRISMA model review was conducted across four databases: PubMed, Science Direct, Google Scholar, and Scopus. Medical Subject Heading (MeSH) terms and keywords (vape, e-cigarette, smoking, social media, influencer, adolescent) were used to search for full-text studies published in English from the last five years. The studies included focused on human subjects aged 10-21 and met predefined eligibility criteria.

Of the 1,064 articles initially identified, 37 were included after screening and removing duplicates.

**Results:**

Thirty-seven articles met the eligibility criteria. The analysis focused on five key social media platforms: TikTok, Instagram, YouTube, Twitter, and Facebook. However, significant methodological limitations were noted in the studies.

**Conclusions:**

The research highlights a strong link between social media use and youth e-cigarette consumption. Exposure to vaping content, ads, and peer influence on social media increases the risk, particularly among younger and vulnerable groups. Social media enables both direct marketing and peer-to-peer promotion. Stricter regulations on online ads and enhanced parental controls are necessary to mitigate these risks and reduce youth vaping rates.

**Disclosure of Interest:**

None Declared

